# *IL10A* genotypic association with decreased IL-10 circulating levels in malaria infected individuals from endemic area of the Brazilian Amazon

**DOI:** 10.1186/s12936-015-0548-z

**Published:** 2015-01-28

**Authors:** Virginia A Pereira, Juan C Sánchez-Arcila, Antonio Teva, Daiana S Perce-da-Silva, Mariana PA Vasconcelos, Cleoni AM Lima, Cesarino JL Aprígio, Rodrigo N Rodrigues-da-Silva, Davi O Santos, Dalma M Banic, Maria G Bonecini-Almeida, Josué C Lima-Júnior, Joseli Oliveira-Ferreira

**Affiliations:** Laboratório de Imunoparasitologia, Instituto Oswaldo Cruz/Fiocruz, Av. Brasil 4365, Manguinhos, Rio de Janeiro Brazil; Laboratório de Imunodiagnóstico /Departamento de Ciências Biológicas, Escola Nacional de Saúde Pública/Fiocruz, Rio de Janeiro, Rio de Janeiro Brazil; Laboratório de Simulídeos e Oncocercose, Instituto Oswaldo Cruz/Fiocruz, Rio de Janeiro, Brazil; Instituto de Infectologia Emilio Ribas, São Paulo, São Paulo Brazil; Centro Interdepartamental de Biologia Experimental e Biotecnologia, Universidade Federal de Rondônia, Porto Velho, Rondônia Brazil; Laboratório de Quimioterapia/Fiocruz, Porto Velho, Rondônia, Brazil and Universidade Federal de Rondônia, Porto Velho, Rondônia Brazil; Laboratório de Imunologia e Imunogenética, Instituto de Pesquisa Clínica Evandro Chagas (IPEC)/Fiocruz, Rio de Janeiro, Rio de Janeiro Brazil

**Keywords:** Malaria disease, Cytokines, Nitric oxide synthase, Polymorphism

## Abstract

**Background:**

Cytokines play an important role in human immune responses to malaria and variation in their production may influence the course of infection and determine the outcome of the disease. The differential production of cytokines has been linked to single nucleotide polymorphisms in gene promoter regions, signal sequences, and gene introns. Although some polymorphisms play significant roles in susceptibility to malaria, gene polymorphism studies in Brazil are scarce.

**Methods:**

A population of 267 individuals from Brazilian Amazon exposed to malaria was genotyped for five single nucleotide polymorphisms (SNPs), *IFNG + 874 T/A*, *IL10A-1082G/A*, *IL10A-592A/C*, *IL10A-819 T/C* and *NOS2A-954G/C*. Specific DNA fragments were amplified by polymerase chain reaction, allowing the detection of the polymorphism genotypes. The polymorphisms *IL10A-592A/C* and *IL10A-819 T/C* were estimated by a single analysis due to the complete linkage disequilibrium between the two SNPs with D’ = 0.99. Plasma was used to measure the levels of IFN-γ and IL-10 cytokines by Luminex and nitrogen radicals by Griess reaction.

**Results:**

No differences were observed in genotype and allelic frequency of *IFNG + 874 T/A* and *NOS2A-954G/C* between positive and negative subjects for malaria infection. Interesting, the genotype NOS2A-954C/C was not identified in the study population. Significant differences were found in *IL10A-592A/C* and *IL10A-819 T/C* genotypes distribution, carriers of IL10A -592A/-819 T alleles (genotypes AA/TT + AC/TC) were more frequent among subjects with malaria than in negative subjects that presented a higher frequency of the variant C allele (p < 0.0001). The presence of the allele C was associated with low producer of IL-10 and low parasitaemia. In addition, the GTA haplotypes formed from combinations of investigated polymorphisms in *IL10A* were significantly associated with malaria (+) and the CCA haplotype with malaria (−) groups. The *IL10A-1082G/A* polymorphism showed high frequency of heterozygous AG genotype in the population, but it was not possible to infer any association of the polymorphism because their distribution was not in Hardy Weinberg equilibrium.

**Conclusion:**

This study shows that the *IL10A-592A/C* and *IL10A-819 T/C* polymorphisms were associated with malaria and decreased IL-10 levels and low parasite density suggesting that this polymorphism influence IL-10 levels and may influence in the susceptibility to clinical malaria.

## Background

Malaria is an infectious disease that affects millions of people each year worldwide. The species of *Plasmodium* that affect humans have different pathogenic potential. However, beyond the pathogenic potential of the parasite, there are environmental factors, host genetics and parasite virulence associated to susceptibility and resistance to malaria [1,2]. The identification of host factors may increase the understanding of the interactions between the parasite and host, as well as the mechanisms involved in the pathology and immunity. In human malaria, a link between enhanced IFN-γ, TNF, IL-6, IL-10 and nitric oxide (NO) levels and severity of the disease have long been reported [3-7], although this is not a consistent finding [8-10]. In recent years, several studies have demonstrated that the presence of polymorphisms in IFN-γ, IL-10 and NO gene have been associated with susceptibility or resistance to various diseases [11-15].

The main polymorphism in the gene encoding IFN-γ (*IFNG + 874 T/A* polymorphism) is located in its first intron at position +874 and studies have reported only weak associations between *IFNG* SNPs and susceptibility to severe malaria [16,17]. In Brazil, there is one study showing that *IFNG + 874 T/A* polymorphism are associated with reduced levels of IFN-γ in patients with the homozygote mutant AA genotype while carriers of the wild alleles (AT and TT) were associated with higher levels of this cytokine [18].

IL-10 cytokine has an important regulatory role and polymorphisms in the promoter region of *IL10A* impair the production of this cytokine [19,20], and may contribute to the pathogenesis of diseases. In malaria, the role of IL-10 in regulating the inflammatory response remain conflicting since several studies suggest that enhanced IL-10 is associated with increased pathogenesis while others associate with protection [10,21-23]. The coding gene of IL-10 cytokine contains a promoter region with at least 5 kb, which were described over 27 polymorphisms [19]. In malaria, polymorphisms in the promoter region (*IL-10A-1082A/G*, −*819 T/C* and *-592A/C*) were associated with reduced IL-10 plasma levels and with the development of acute anaemia in Kenyan children with *P. falciparum* malaria in holoendemic areas [20].

Nitric oxide synthase 2 is the critical enzyme involved in the synthesis of nitric oxide (NO), a molecule with diverse functions. There has been much speculation about the part played by nitric oxide in malaria, both as an antiparasitic agent and as a potential cause of cerebral malaria [24-27]. A report from Gabon suggests that a single nucleotide polymorphism in the inducible nitric oxide synthase (*NOS2A*) promoter is associated with protection from all forms of severe malaria, including susceptibility to reinfection while other study report an association with the risk of fatal cerebral malaria [28]. Recently, an association was found between mutation of a nucleotide at position 84 in the gene of the enzyme, *NOS2A* and a higher risk of cerebral malaria [29]. Moreover, associations between protection against severe malaria and polymorphic forms of the promoter region in African children have also been described [26]. However, few studies evaluate the effect of the gene polymorphism in the promoter of the *NOS2A* gene in NO production. Although some polymorphisms play significant roles in susceptibility to malaria, several findings are inconclusive and contradictory and studies that explore the influence of these polymorphisms in Brazil is scarce [18,30-32]. Thus, cytokine gene polymorphisms have an unquestionable role in the orchestration of the immune response, leading to different functional scenario, which in turn influence the outcome of disease establishment and evolution.

The hypothesis is that SNP polymorphisms may result in changes in recognition sites of some transcription factors that influence the levels of pro-and anti-inflammatory cytokines in malaria infection and may lead to imbalance between these molecules that could favor the host susceptibility to *Plasmodium* and increase the risk for clinical malaria in individuals naturally exposed to infections. Therefore, this study examined the SNPs polymorphisms that affect the expression of genes encoding IFN-γ (−874 T/A), IL-10 (−1082A/G, −819 T/C and -592A/C) and iNOS (−954G/C) from a Brazilian Amazonian population living in malaria endemic area of Brazil.

## Methods

### Subjects and methods

The present study included 267 individuals (malaria-exposed group) from Porto Velho, Rondônia State, malaria endemic area in the southwestern Brazilian Amazon. Among these individuals 73 (27.3%) were positive for malaria infection (malaria (+) group) and 194 (72.7%) individuals living in the same area were negative for malaria infection (malaria (−) group). Malaria diagnosis was performed in Giemsa-stained thin and thick blood smears and parasitological evaluation was done by examination of 200 fields at 1,000X magnification under oil-immersion. The parasitaemia was expressed as the number of parasites/μl of blood in the thick blood smear. The number of parasites/μl of blood was calculated by multiplying the number of parasites counted against 500 leucocytes, and the number of leukocytes of the subject and dividing the product by 500. A researcher expert in malaria diagnosis examined all slides. To confirm the parasitological diagnosis, molecular analyses of all samples was performed using primers specific for genus (*Plasmodium* sp.) and species (*Plasmodium falciparum* and *Plasmodium vivax*). The amplification protocols were described previously by Snounou *et al.* [33]. Subjects were considered to have malaria if they were positive in the thick blood smear and/or polymerase chain reaction (PCR). Positive volunteers for *P. vivax* and/or *P. falciparum* at the time of blood collection were subsequently treated using the chemotherapeutic regimen recommended by the Brazilian Ministry of Health [34]. Written informed consent was obtained from all volunteers and the study was reviewed and approved by the Fundação Oswaldo Cruz Ethical Committee and the Brazilian National Ethical Committee. All volunteers were clinically evaluated and answered an epidemiological questionnaire, including data as age, gender, time of residence in endemic area, number of past infections, past and last *Plasmodium* species infection and time since last infection.

### DNA extraction and genotyping

**Figure 1:**
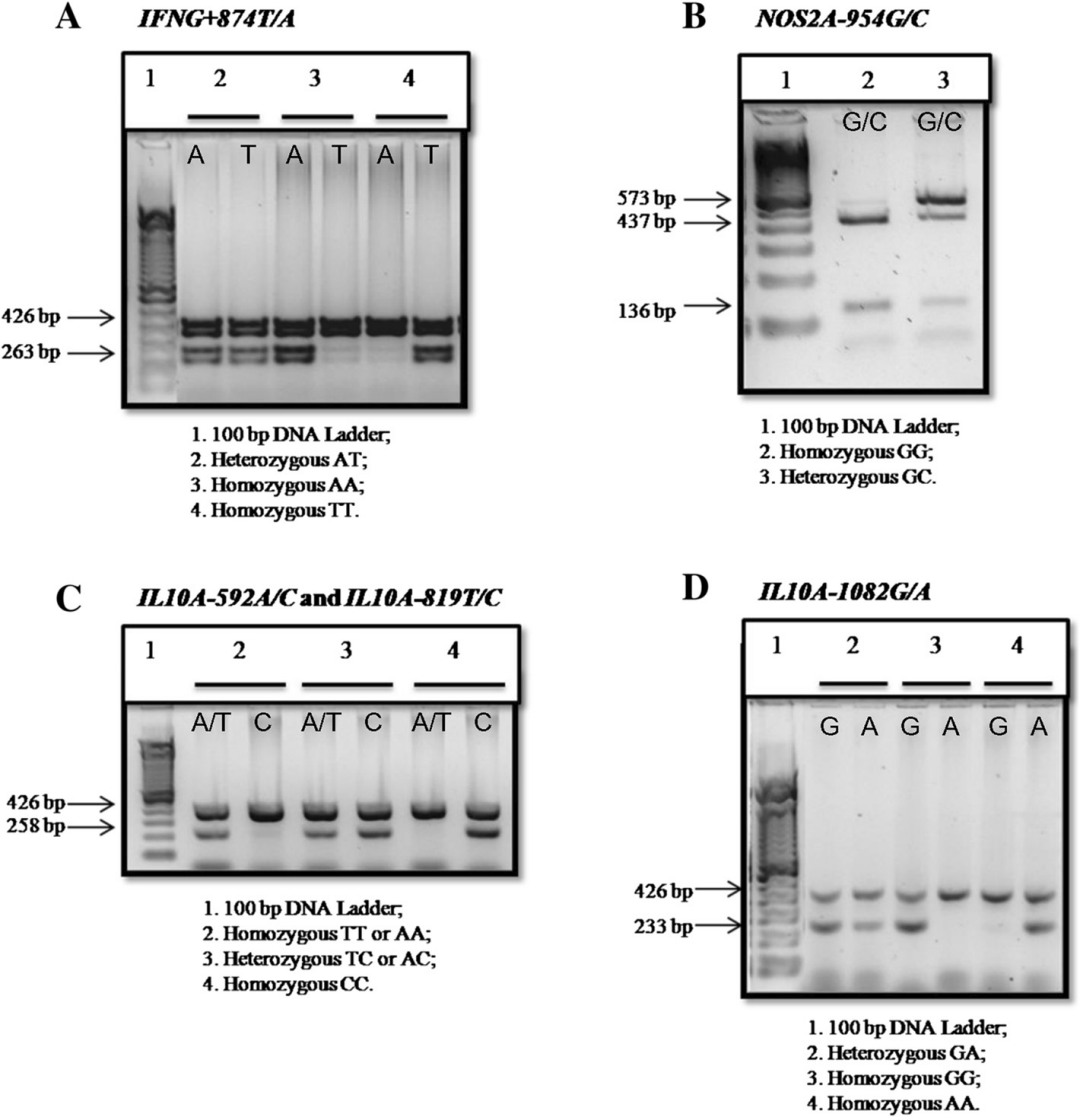
**Visualization of amplified fragments on agarose gel.** 426 bp fragment: band corresponding to internal control using a pair of primers designed from the nucleotide sequence of human growth hormone; **A.** 263 bp fragment: specific bands for T and A alleles of *IFNG + 874 T/A* polymorphism; **B.** 437 and 136 bp fragments: specific bands for G allele of *NOS2A-954G/C* polymorphism; 573 bp fragment: specific band for C allele of *NOS2A-954G/C* polymorphism; **C.** 258 bp fragment: specific bands for A/T and C alleles of *IL10A-592A/C* and *IL10A-819 T/C* polymorphisms were estimated by a single analysis due to the complete linkage disequilibrium of the two SNPs (D’ = 0.99), **D.** 233 bp fragment: specific bands for G and A alleles of *IL10A-1082G/A* polymorphism.

Genomic DNA was extracted using the kit QIAamp® DNA Blood Midi/Maxi (QIAgen, Hilden, Germany), quantified using the NanoDrop ND-1000 and stored at −20°C until use. Amplification Refractory Mutation System (ARMS-PCR), first described by Newton et *al.* [35], analysed single nucleotide polymorphisms (SNPs) for the IFNG + 874 T/A, IL-10A-1082A/G, IL10A-819 T/C and IL10A-592A/C, and *NOS2A-954G/C* polymorphism by Restriction fragment length polymorphism (RFLP) [26]. The polymorphisms *IL10A-592A/C* and *IL10A-819 T/C* were estimated by a single analysis due to the complete linkage disequilibrium between the two SNPs with D’ = 0.99. Amplifications were performed in a GeneAmp PCR System 9700 (Applied Biosystems, Foster City, CA) using 2.5UI for *IFNG* and *IL10A* and 1.5UI for *NOS2A* of Taq DNA polymerase (5U/ μL, Invitrogen). Cycling PCR conditions for *IFNG + 874 T/A* were 95°C (3 minutes), 10 cycles of 95°C (15 seconds), 65°C (50 seconds), 72°C (40 seconds) followed by 20 cycles of 95°C (20 seconds), 55°C (50 seconds) and 72°C (50 seconds), 72°C (7 minutes) 4°C until use. Cycling PCR conditions for *IL10A* polymorphisms were 95°C (1 minute), 10 cycles of 95°C (15 seconds), 65°C (50 seconds), 72°C (40 seconds) followed by 20 cycles of 95°C (20 seconds), 59°C (50 seconds) and 72°C (50 seconds), 4°C until use. Cycling PCR conditions for *NOS2A-954G/C* were 95°C (3 minutes), 30 cycles of 94°C (10 seconds), 60°C (30 seconds), 72°C (30 seconds); 72°C (7 minutes) and 4°C until use. The NOS2A-954G/C amplified product was subsequently digested with BSAI restriction enzyme in the following condition: 50°C (60 minutes), 65°C (20 minutes) and 4°C until use. All amplified products were evaluated by electrophoresis on a 1.5% (*IFNG* and *IL10A*) and 2.5% (*NOS2A*) agarose gel containing ethidium bromide (0.5 μg/mL). After electrophoresis, the fragments were displayed and the images were photographed on a transilluminator Multi Doc-It™ Digital Imaging System (UVP, Upland, CA) (Figure 1).

### Analysis of plasma cytokines concentration

Levels of IFN-γ and IL-10 were detected in plasma samples by a multiplex assay (Bio-Plex assay, Bio-Rad Laboratories, Hercules, CA, USA) according to the manufacturer’s instructions using the Luminex system (Luminex Corporation, Austin, TX, USA) and analysed with a Bio-Plex suspension array system (Bio-Rad Laboratories). Fluorescence intensity was transformed into cytokine concentration using the Bio-Plex manager software (version 3.0). A minimum of 100 beads per region were analysed. A curve fit was applied to each standard curve according to the manufacturer’s manual and sample concentrations were interpolated from the standard curves. The limit of detection was 0.79 pg/mL for IFN-γ and 2 pg/mL for IL-10.

### Griess microassay detection of nitrite and nitrate

A modified Griess reaction was used to detect nitrite and nitrate [36,37]. The NO levels in samples were indirectly measured after first converting nitrates to nitrites with a nitrate reductase treatment (*Aspergillus* species NAD [P] H, Sigma, UK) and NADPH β-nicotinamide adenine dinucleotide phosphate (Sigma Diagnostics, St. Louis, USA). Griess reagent [5% phosphoric acid, 1% sulphanilic acid and 0.1% N-(1-naphthyl-1)-ethylendiaminedihydrochloride, all from Sigma, UK, dissolved in 100 mL deionized water] was added and proteins were subsequently precipitated by trichloroacetic acid (BDH, England). The tube contents were mixed and centrifuged (Eppendorf centrifuge 5415 C, Germany); two samples of each supernatant were transferred to a flat-bottomed microplate and their absorbances were read at 520 nm using a microplate reader (SpectraMax, Molecular Devices Inc). NO values were calculated from standard calibration plots.

### Statistical analysis

Epidemiological and experimental data were stored in the Epi- Info 3.5.1 (CDC, Atlanta, USA). Allele and genotype frequencies were estimated by gene counting and differences between groups by χ^2^ test. The risk of malaria associated with polymorphisms was estimated using odds ratios (OR) and confidence interval of 95% (CI) with and without adjustment by age, gender, length of residence in an endemic area and number of previous episodes of malaria. Differences between malaria (+) and malaria (−) groups were estimated by Mann–Whitney and t tests. Relationships between epidemiological factors and cytokine levels were assessed by Spearman’s correlation The Hardy-Weinberg equilibrium (HWE) was assessed by χ^2^ test and Fisher Person. The genetic analyses correspond to codominant logistic regression model (homozygous major allele VS. Heterozygote + homozygote secondary). The linkage disequilibrium was calculated by D statistic and frequency haplotypes by calculating the maximum likelihood estimator via the EM algorithm (Expectation Maximization) using two-step, step “E” (Hope) and step “M” (max) until convergence is achieved. Statistical analyses were performed using Graph Pad Prism software and SNPStats 5.0 (San Diego, California, USA). Statistically significant values of p < 0.05 was considered.

## Results

### Description of the studied population

The epidemiological surveillance shows that a significant proportion (93.16%) of the inhabitants reported a prior infection with *P. vivax* and/or *P. falciparum* indicating that the majority of the individuals were exposed to malaria parasite throughout the years. Among studied participants, 55.4% were male and the mean age was 30.99 ± 14.37 years. Comparing both groups, a higher frequency of males was observed in malaria (+) group. However, no statistical differences were identified regarding age, the length of residence in an endemic area and number of previous episodes of malaria between groups. Yet, the time since last infection was higher in malaria (−) group (Table 1). In malaria (+) group, 53 (72.6%) individuals were infected with *P. vivax* and 20 (27.4%) with *P. falciparum*, consistent with *Plasmodium* species distribution reported by the Brazilian Ministry of Health [34]. No cases of severe malaria were seen and fever and headache were the main reported symptoms in all patients. Parasitaemia and symptoms were similar in patients infected with both plasmodial species (Table 2).

**Table 1:** **Characteristics of the study participants**

	**Malaria diagnosis**			
	**Positive N = 73**	**Negative N = 194**	**Total N = 267**	*P **value**
**Gender n (%)**				
♀	21 (28.8%)	98 (50.5%)	119 (44.6%)	0.001
♂	52 (71.2%)	96 (49.5%)	148 (55.4%)	0.001
**Age**	28 (9–54)	30 (4–71)	29 (4–71)	0.531
**TREA (years)**	25 (1–33)	24 (2–32)	24 (1–63)	0.079
**NPE**	5 (0–50)	5 (0–99)	5 (0–99)	0.072
**TLI (Months)**	6 (0–360)	12.5 (0–420)	12 (0–420)	0.016

Data expressed as n (%) of patients; Time of Residence in years TREA; Number of Previous Episodes NPE; Time since Last Infection TLI; Age, TREA, NPE and TLI expressed as median (minimum-maximum); *Statistical significance determined by Mann Whitney U and Chi-square tests.

**Table 2:** **Malaria symptoms and parasitaemia according to plasmodial species**

**Plasmodial species (n = 73)**				
	***P. vivax***	***P. falciparum***	**Total**	*P **value**
**Number of infected n (%)**	53 (72.6%)	20 (27.4%)	73	<0.0001
**Parasitaemia**	2286 (50–17933)	1214 (52–12623)	2097 (50–17933)	0.334
**Days since the onset of symptoms**	2 (1–5)	3 (1–10)	3 (1–10)	
**Fever**	38 (71.7%)	15 (75%)	53 (72.6%)	0.779
**Headache**	40 (75.5%)	15 (75%)	55 (75.3%)	0.968
**Chill**	32 (60.4%)	13 (65%)	45 (61.6%)	0.719
**Myalgia**	31 (58.5%)	14 (70%)	45 (61.6%)	0.368
**Nausea**	20 (37.7%)	11 (55%)	31 (42.5%)	0.184

Data expressed as n (%) of patients; Parasitaemia (number of parasites/μL of blood) and onset of symptoms expressed as median (minimum-maximum); *Statistical significance determined by Mann Whitney U and Chi-square tests.

### IFN-γ, IL-10 and NO plasma levels

**Figure 2:**
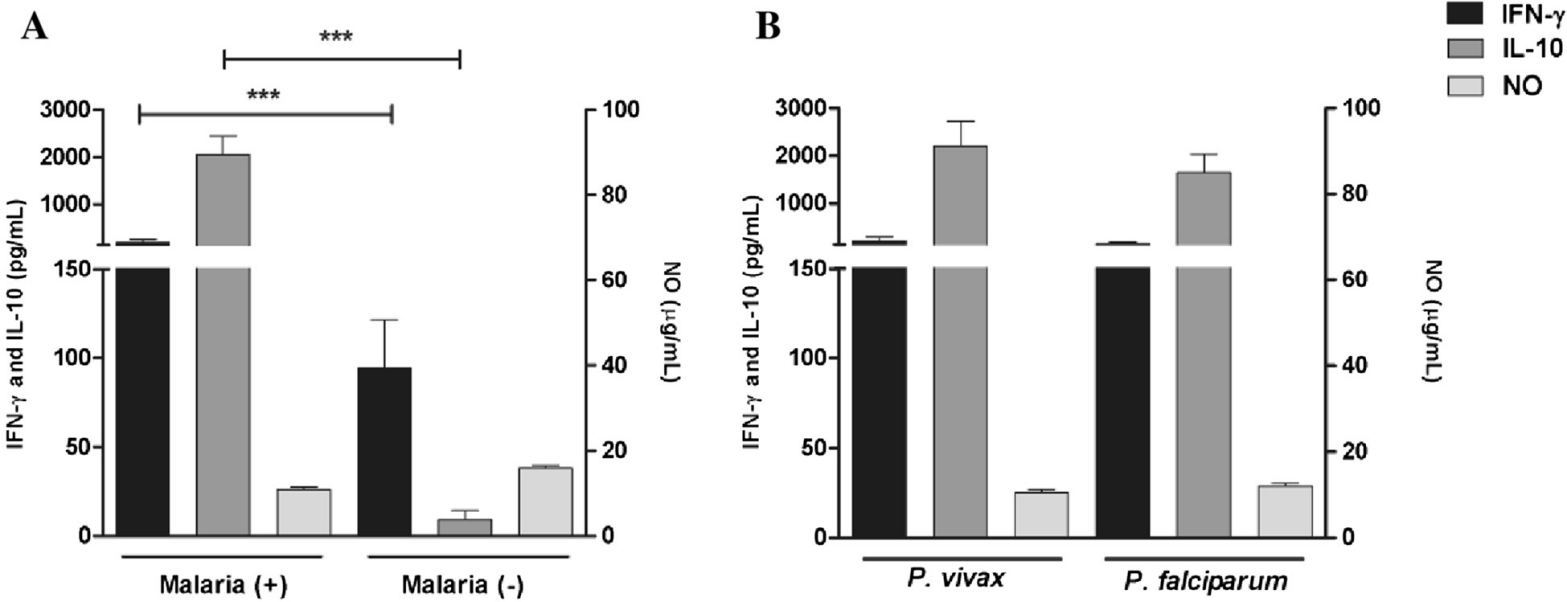
**Levels of IFN-γ (pg/mL), IL-10 (pg/mL) and NO (μg/mL) were compared in Malaria (+) and Malaria (−) groups and according to the plasmodial species.** Levels of IFN-γ, IL-10 and NO in: **A**. Malaria (+) and Malaria (-) groups and **B**. *Plasmodium vivax* and *Plasmodium falciparum* infections. Data are expressed as means levels (means ± SEM) on a logarithmic scale; Statistical significance determined by Mann Whitney U test; ***P value < 0.0001.

**Figure 3:**
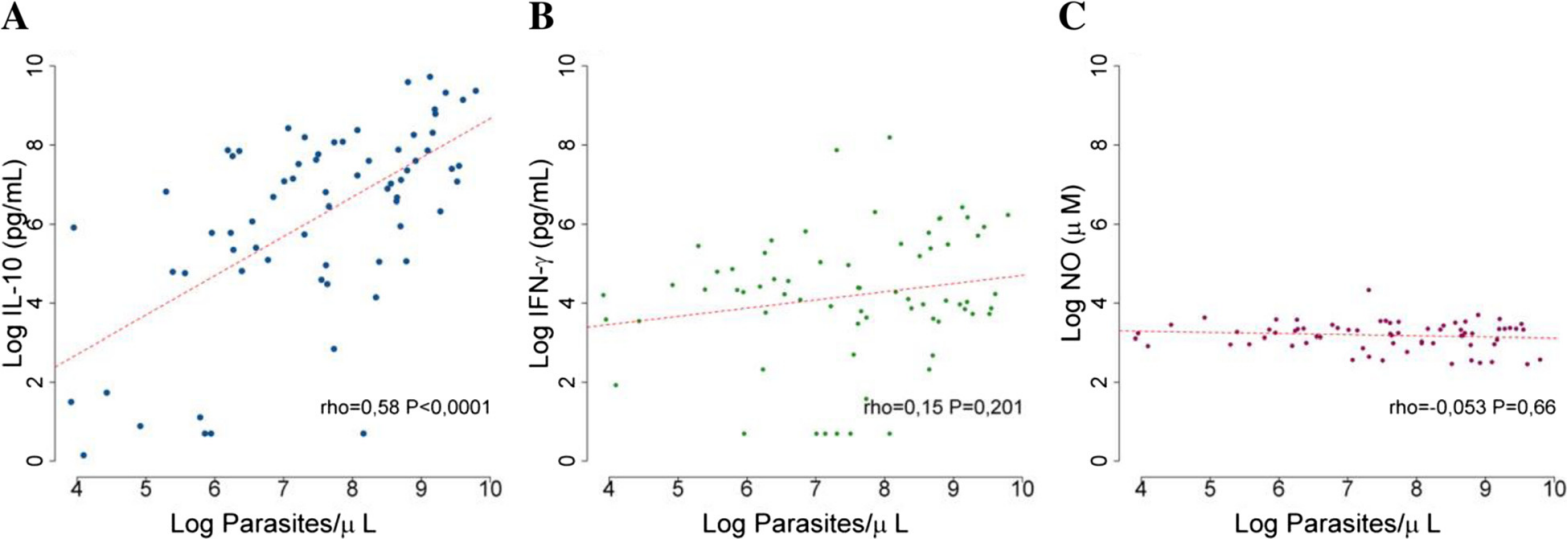
**Correlation of cytokines and nitrogen radicals levels with parasite density in malaria patients. A**. IL-10 levels, **B**. IFN-γ levels and **C**. NO levels. High titers of IL-10 serum levels were correlated with parasitaemia by Spearman correlation (rho = 0.58, p < 0.0001).

The means levels of IFN-γ (66.71 pg/mL [2.00-3629.00]) and IL-10 (795.34 pg/mL [1.15-16782.93]), were higher in plasma of malaria (+) when compared with malaria (−) group (IFN-γ 19.44 pg/mL [1.46-3919.00] and IL-10 1.29 pg/mL [0.40-989.00]). The levels of NO did not differ significantly when the groups were compared (malaria (+) 25.49 μM [11.65-75.85] and malaria (−) 35.41 μM [0.52-110.95]) (Figure 2A). No differences were observed in the levels of IFN-γ (58.96 pg/mL [2–3629]), IL-10 (627.39 pg/mL [1.15-16782.9]) and NO levels (23.98 μM [11.65-75.85]) in *P. vivax* (n = 53) infected individuals when compared with the levels of IFN-γ (98.40 pg/mL [2.00-548.15]), IL-10 (1149.88 pg/mL [3.02-6540.45]) and NO levels (28.49 μM [12.99-40.31]) in *P. falciparum* (n = 20) infected individuals (Figure 2B). Although both IL-10 and IFN-γ were higher in malaria (+) group, Figure 3 shows that the correlation between cytokine levels and parasite density were only observed for IL-10 (rho = 0.58 p < 0.0001).

### Alleles, genotypes and haplotypes frequencies obtained for IFNG, IL10A and NOS2A genes

The SNPs investigated, their location in the gene, the genotype and allele frequencies observed in malaria (+) and malaria (−) groups are presented in Table 3. The groups fell within Hardy-Weinberg equilibrium with non-significant values by χ^2^ test for the genotype observed and expected for all polymorphism except for the *IL10A-1082G/A* (χ^2^ = 41.76, p *<* 0.0001, χ^2^ = 32.33, p *<* 0.0001). The *IFNG + 874 T/A* genotype and allelic frequencies did not differ between malaria (+) and malaria (−) groups. In both groups, the most frequent genotype for *IFNG + 874 T/A* was the homozygous mutant AA followed by AT heterozygous and homozygous wild T allele. The variant allele +874 A was more frequent than wild T. In contrast, the distribution of the *IL10A-1082G/A* genotype differed between malaria (+) and malaria (−) groups while allelic frequencies were similar in both groups. Although the heterozygous AG genotype were more frequent in both groups, this genotype was significantly higher in malaria (+) (p < 0.001). The polymorphisms *IL10A-592A/C* and *IL10A-819 T/C* were estimated by a single analysis due to the complete linkage disequilibrium between the two SNPs with D’ = 0.99. The A allele of *IL10A-592A/C* was always linked with the T allele of and *IL10A-819 T/C* and C with C. IL10A-592A/A and IL10A-819 T/T genotypes were more frequently observed in malaria (+) compared with malaria (−) group (p < 0.001), even when adjusted by sex and age. Similarly the wild alleles A/T were more frequent in malaria (+) than malaria (−) groups (OR = 0.45; p < 0.0001). Stratification of the individuals into haplotypic groups based on the tree promoter *IL10A* polymorphisms at positions −592/-819/-1082 yielded the following haplotypes distribution: Hap1 (CCG), Hap2 (ATA), Hap3 (CCA) and Hap4 (ATG) with frequencies that varied from 7% to 38% in both malaria (+) and malaria (−) groups. As shown in Table 4, Hap1 and Hap2 were the most frequent haplotypes with similar distribution in the groups. The distribution of the haplotypes Hap3 and Hap4 were significantly different between malaria (+) and malaria (−) groups, while the Hap1 and Hap2 were similar between the groups. The Hap3 haplotype was more frequent in malaria (−) while the Hap4 were more frequent in malaria (+) group. The distribution of *NOS2A-954G/C* genotype in the population showed that the homozygous GG was more frequent in both groups and the genotype *NOS2A-954C/C* was not identified in the study population. The G allele was also the most frequent allele with similar distribution in both groups.

**Table 3:** **Genotypic and allelic frequencies of single nucleotide polymorphisms in malaria (+) and malaria (−) groups**

***Studied polymorphisms***		**Malaria (+) (n = 73)**	**Malaria (−) (n = 194)**	**OR (95% CI)**	* ***P value***
***IFNG + 874 T/A***					
Genotypes	A/A	38 (52%)	101 (52.1%)	1.00	0.92
	A/T	30 (41.1%)	77 (39.7%)	0.97 (0.55-1.70)	
	T/T	5 (6.8%)	16 (8.2%)	1.20 (0.41-3.51)	
Alleles	A	106 (73%)	279 (72%)	0.16 (0.68 - 1.58)	0.87
	T	40 (27%)	109 (28%)		
***NOS2A-952G/C***					
Genotypes	G/G	70 (95.9%)	183 (94.3%)	1.00	0.6
	G/C	3 (4.1%)	11 (5.7%)	1.40 (0.38-5.18)	
	C/C	-	-	-	
Alleles	G	143 (98%)	377 (97%)	1.39 (0.38 - 5.05)	0.6
	C	3 (2%)	11 (3%)		
***IL10A-1082G/A***					
Genotypes	A/A	3 (4.1%)	40 (20.6%)	1.00	0.0009
	G/A	64 (87.7%)	135 (69.6%)	0.16 (0.05-0.53)	
	G/G	6 (8.2%)	19 (9.8%)	0.24 (0.05-1.05)	
Alleles	A	70 (48%)	215 (62%)	0.74 (0.50 - 1.08)	0.12
	G	76 (52%)	173 (45%)		
***IL10A-592A/C*** **and** ***IL10A-819 T/C***					
Genotypes	C/C	13 (17.8%)	70 (36.1%)	1.00	0.0002
	A/C; T/C	36 (49.3%)	100 (51.1%)	0.52 (0.26-1.04)	
	A/A; T/T	24 (32.9%)	24 (12.4%)	0.19 (0.08-0.42)	
Alleles	C	62 (42%)	240 (62%)	0.45 (0.30 - 0.67)	0.0001
	A/T	84 (58%)	148 (38%)		

*IFNG + 874 T/A*, *NOS2A-954G/C*, *IL10A-1082G/A*, *IL10A-592A/C* and *IL10A-819 T/C* polymorphisms: Alleles, n (%); Genotypes n (%); OR (95% CI), calculating odds ratios with confidence interval (CI) of 95%; *Analysis by χ^2^ test using the Fisher model codominance.

**Table 4:** **Haplotypes frequencies of** ***IL10A*** **in malaria (+) and malaria (−) groups**

***Haplotypes frequencies***							
	**−592**	**−819**	**−1082**	**Malaria (+)**	**Malaria (−)**	**OR (95% CI)**	* ***P value***
***Hap1***	C	C	G	0.3257	0.37	1.00	-
***Hap2***	A	T	A	0.3805	0.3056	0.85 (0.40 – 1.77)	0.66
***Hap3***	C	C	A	0.0989	0.2486	0.36 (0.14 – 0.93)	0.036
***Hap4***	A	T	G	0.1948	0.0759	3.83 (1.40 – 10.45)	0.009

-592/-819/-1082: Positions of polymorphisms in the promoter region of the interleukin 10 gene; OR (95% CI), calculating odds ratios with confidence interval (CI) of 95%; *Analysis by calculating EMV via EM algorithm using a logistic regression model.

### Association between IFNG, IL-10 and NOS2A genotypes and their products

**Figure 4:**
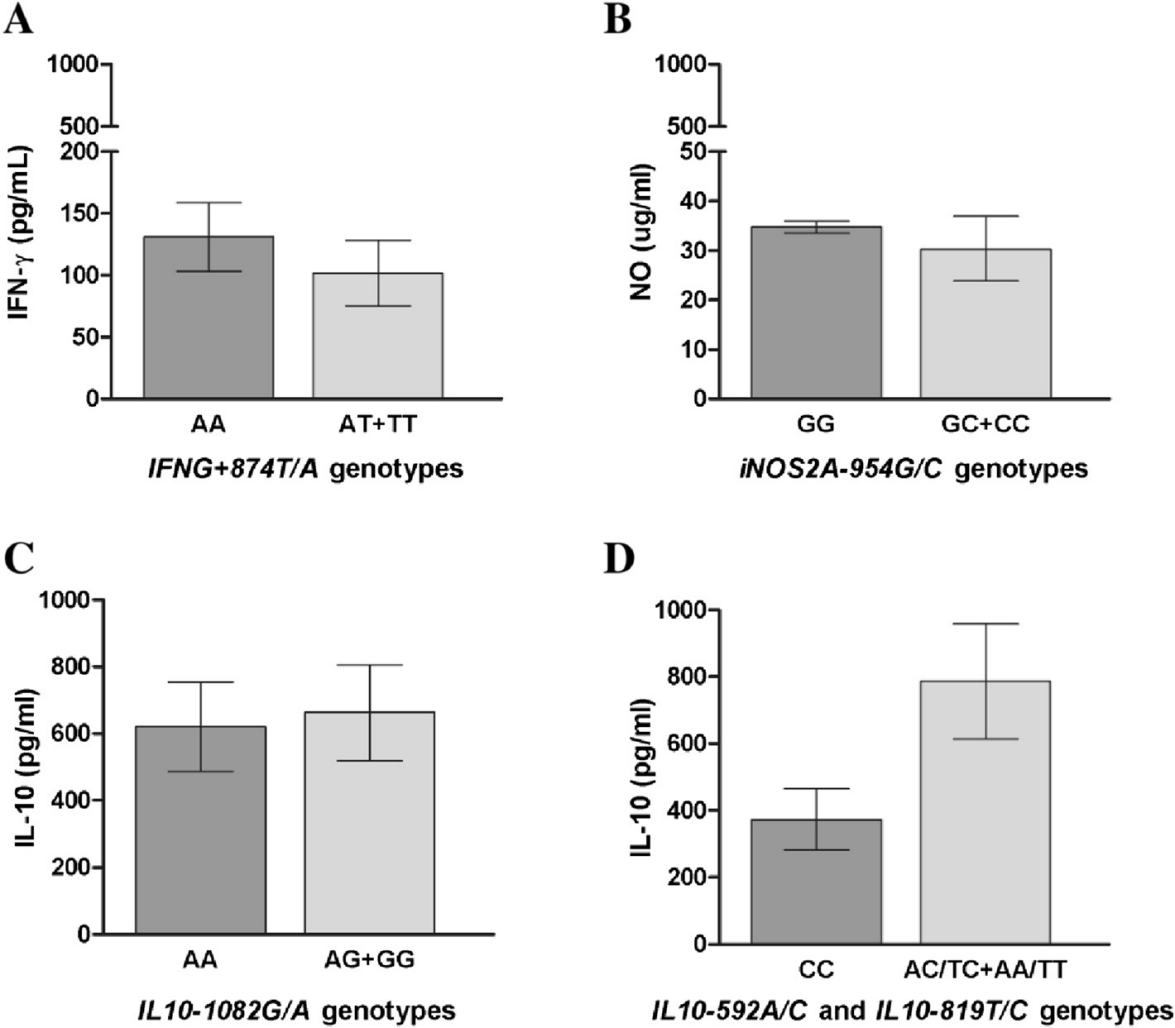
**IFN-γ, IL-10 and nitrogen radicals levels by different alleles carried of polymorphisms. A**. IFN-γ levels in *IFNG-874 T/A genotypes,* **B**. *NO levels in NOS2A-954G/C genotypes,* **C**. *IL-10 levels in IL10A-1082G/A genotypes,* **D**. *IL-10 levels in IL10A-592A/C* and *IL10A-819 T/C* genotypes. Data are expressed as means levels (means ± SEM) on a logarithmic scale; Statistical significance determined by Mann Whitney U test; **P value < 0.001.

No association was observed between *IFNG-874 T/A*, *NOS2A-954G/C* and *IL10A-1082G/A* SNPs and the levels of their products in plasma (Figure 4A-C). However, individuals with *IL10A-592A/C* and *IL10A-819 T/C* genotypes were strongly associated with the plasma levels of IL-10. The IL-10 levels were lower in subjects who carried the homozygous variant IL10A-592CC and -819CC (1.19 pg/mL [0.4-2241]) compared to subjects with IL10A-592 AC and -819TC (2.3 pg/mL [0.4-11233]) and wild homozygous individuals with IL10A-592AA/-819TT (18.4 pg/mL [0.4-16782]) (p < 0.0001). Wild homozygous individuals with IL10A-592AA/-819TT presented IL-10 levels three times higher than individuals carrying variant allele C, indicating an association between the AA/TT genotypes with clinical malaria risk (Figure 4D).

### Polymorphisms and parasitaemia levels association

**Figure 5:**
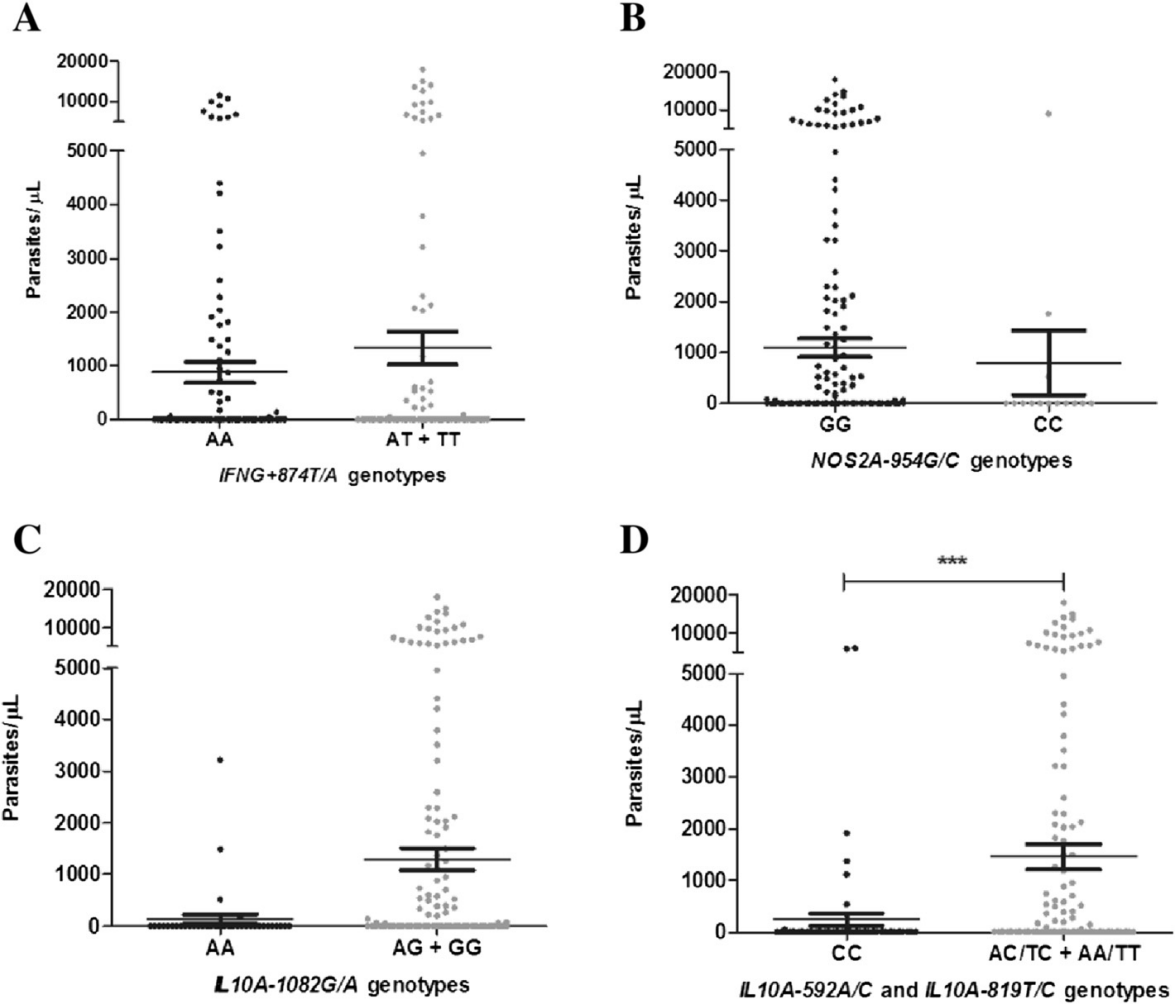
**Parasite density and allelic carried frequencies of polymorphisms.** Parasitaemia levels in: **A**. *IFNG-874 T/A genotypes*, **B**. NOS2A-954G/C genotypes, **C**. IL10A-1082G/A genotypes, **D**. IL10A-*592A/C* and *IL10A-819 T/C* genotypes. Data are expressed as means levels (means ± SEM) on a logarithmic scale; Statistical significance determined by Mann Whitney U test; **P value < 0.001.

The parasite densities among the different genotypes and carried alleles were evaluated and the parasitaemia was not influenced by the *IFNG + 874 T/A*, *NOS2A-954G/C* and *IL10A-1082G/A* polymorphisms (Figure 5). Among the *IFNG + 874 T/A* genotypes, the median parasitaemia was 37.85 (50–17933) parasites/μL for heterozygous genotype AT, 11.72 (199–6518) parasites/μL for homozygous wild TT and 1861 (52–11546) parasites/μL for homozygous variant AA (Figure 5A). Among the *NOS2A-954G/C* polymorphism, the homozygous CC variant was not detected and the median parasite density was 1758 (524–8888) parasites/μL for GC genotype and 2120 (50–17933) parasites/μL for homozygous GG (Figure 5B). The *IL10A-1082G/A* genotypes, the median parasitaemia was 1485 (510–3210) parasites/μL for the homozygous AA variant, 2200 (50–17933) parasites/μL for the heterozygous AG and 1906 (84–6000) parasites/μL for homozygous GG wild (Figure 5C). The IL10A-592AA and IL10A-819TT genotypes presented 2440 (52–17933) parasites/μL while the heterozygous IL10A-592 AC and IL10A-819TC presented 2280 (84–14891) parasites/μL (Figure 5D). These genotypes (carriers of the wild type) presented higher parasites densities when compared with the homozygous variant IL10A-592CC and IL10A-819CC 1108 (50–6000) parasites/μL.

## Discussion

Human populations display differences in susceptibility to many diseases and the basis for this differential susceptibility is, at least in part, genetically determined [38,39]. Significant associations between cytokine polymorphism and diseases support that cytokine gene polymorphisms have an unquestionable role in the orchestration of the immune response, leading to different functional scenario, which in turn influence the outcome of disease establishment and evolution [38,40]. Thus, the present study aimed at exploring cytokine and NO polymorphism in populations naturally exposed to malaria, residents in Rondônia State, southwestern of Brazilian Amazon in order to establish the possible implications of these polymorphisms in malaria infection as well as analysing whether the alleles and genotypes are associated with their expression. In recent years, evaluation of SNPs have been considered a common approach for testing human genetic variation [37].

The SNPs investigated include *IFNG* (−874 T/A), *IL10A* (−1082A/G, −819 T/C and -592A/C) and *NOS2A* (−954G/C). Firstly, genotypic and allelic frequencies of the *IFNG + 874 T/A* polymorphism showed that the variant allele A was more common in the population as well as the corresponding homozygous AA genotype. The frequencies found in the study population, are in agreement with previous studies conducted in other Brazilian [18] and Colombian Amazon regions populations [41]. However, studies conducted in Brazil in non-Amazonian areas the homozygous AA genotype was predominant in patients with tuberculosis, while the control group of individuals from the same region had the heterozygous genotype predominantly AT, demonstrating a heterogeneous composition in different geographic region of Brazil, beyond the significant association of this polymorphism with tuberculosis [11,13].

In the study population, no difference in the allelic and genotypic distribution of *IFNG + 874 T/A* polymorphism was observed between malaria (+) or malaria (−) groups. Few studies find associations between severe malaria and *IFNG + 874 T/A* polymorphism and these associations were weak and not significant after correction for multiple comparisons [42,43]. In malaria-infected individuals, the relationships between *IFNG + 874 T/A* polymorphism and IFN-γ serum levels were not observed in this study, although high levels of IFN-γ were detected in malaria (+) subjects. The results of this study were different from Medina *et al.* that reported in a population of an endemic area in Brazil, a concentration of IFN-γ significantly lower in the serum of patients with AA individuals compared with T wild allele carriers [18]. The possibility that there exist functional *IFNG* polymorphisms that were not effectively tagged by this marker cannot be excluded, and further studies of the locus are warranted.

In the *IL10A* gene, investigation of three polymorphisms at positions −592, −819 and 1082 of the promoter region, were associated with the actual production of IL-10, which has an important role in the immune response to malaria infection [21,44,45]. The *IL10A* gene polymorphism at position −1082, have been associated with decreased production of IL-10 and clinical and severe malaria [7,20,46]. However, no association was found between this polymorphism and the levels of IL-10 nor with the occurrence of the disease and this polymorphism were not in Hardy-Weinberg equilibrium in the studied population, demonstrating the need for a representative sample to verify a possible association of this polymorphism with IL-10 levels in malaria patients. In the studied population the heterozygous GA was the most frequent genotype in both malaria (+) and malaria (−) groups. Medina *et al.* [18] reported the homozygous AA variant as the most common genotype in the population of Belem (Para state) but showed weak association between IL-10 concentration and parasite density [18], while a study in the Amazonas state revealed an association between *IL-10A-1082G/A* polymorphism with reduced risk to clinical malaria [32]. Some studies that have addressed this polymorphism showed variation in the allele and genotype distribution according to ethnicity [18,20,47]. In the case of the *IL10A-592A/C* and *-819 T/C* polymorphisms, it was observed association with IL-10 production and parasite density. Interestingly, a high prevalence of the C alleles and homozygous variant -592CC/-819CC was found in malaria (−) group while a higher prevalence of wild alleles A/T and homozygous genotype -592AA/-819TT in malaria (+), suggesting that the C allele were at lower risk to have malaria due to its prevalence in the malaria (−) group. The homozygous genotype -592CC/-819CC was associated with a reduced IL-10 levels and low parasite density compared to other genotypes. Indeed, carriers of the C allele variant were low producers of IL-10 and presented low parasite density while carriers of A/T wild alleles were high producer of IL-10 and presented high parasite density. The presence of the CC genotype may create site for enhanced binding of repressor that favor reduced IL-10 production.

Considering the haplotypes of *IL10A* at positions −592/-819/-1082, no association was observed between the most frequent haplotypes CCG and ATA with the risk of having malaria. However, carriers of the less frequent haplotype GTA were more prevalent in malaria (+) group while carriers of the ACC haplotype were more prevalent in malaria (−) group. The haplotype analysis of these three polymorphism in the Amazonas state, the GCT allelic combination were associated with low risk of any form of malaria, this haplotype was not present in the study population in Rondônia State neither in Para State, both Amazonian endemic areas [31]. The differences in haplotypes distribution in the same region are consistent with the heterogeneous genetic profile of Brazilian population [48]. Study in Kenia reported relationship between common Africa *IL10A* promoter variants and protection against severe malarial anaemia and increased production of IL-10 [20]. However, other studies have shown no evidence of association between the polymorphisms in the IL-10 gene and malaria severity [18]. A study in Gambia showed an association between the haplotype of five SNPs (+4949G, +919C, −627G, −1117C, −3585 T), not evaluated in this study, and resistance to cerebral malaria and severe anaemia [49]. In Brazil, this is the first report that investigate the frequency of the promoter region haplotypes in *IL10A* gene associated with malaria infection, IL-10 levels and parasite density. Similar *IL10A* haplotypes distribution were reported in a population from the State of Para another malaria endemic area in Brazil. However, the authors did not evaluate the IL-10 levels and did not find any influence of these haplotypes in susceptibility to malaria [31].

Finally, it was not found association between *NOS2A-954G/C* polymorphism and susceptibility to malaria, NO levels or parasitaemia. Indeed, the G-954-C C allele was present in less than 4% of the study population, it is absent in Caucasian and is found at low frequency in Asia. [26]. In contrast, in African population this genotype is present in high frequency where most of the associations with malaria outcome were reported. Even though, there has been much speculation about the role played by nitric oxide (NO) in malaria, both as an antiparasitic agent and as a potential cause of cerebral malaria [37]. In Brazil, the *NOS2A-954G/C* polymorphism have been reported in studies with tuberculosis and leprosy. In both studies, the allelic and genotypic frequencies were similar to the one found in this study, even though their population were from South and Southeast region [50,51]. Although no association has been found, this study is the first to report *NOS2A-954G/C* polymorphism and NO levels in malaria exposed individuals in endemic region of Brazil. Indeed, the *NOS2A* gene polymorphism have been associated with susceptibility to *P. falciparum* malaria and conflicting results have been obtained in studies that associate the presence of the G-954-C C allele and either risk of cerebral malaria or NO production [26,52]. It should be noted that individual differences in the levels of the cytokines and NO measured at a specific moment may not only result from host genetic factors predisposing to high or low production, but also for a great part from the physiological condition at that time, as well as from general immunity. The present findings reinforce the role of mediators of inflammation in malaria susceptibility and future studies in different setting with large samples numbers are warranted. Furthermore, it should be observed that not merely one genetic alteration but rather the combination of a set of genetic factors might influence the susceptibility or resistance to malaria.

## Conclusions

This study shows that *IFNG + 874 T/A, IL10A-1082G/A* and *NOS2A-954G/C* polymorphisms was not associated with the occurrence of malaria or with the production of its respective cytokine and nitric oxide products. The IL10A-592A/C and IL10A-819 T/C polymorphisms were associated with malaria and decreased IL-10 levels and low parasite density suggesting that this polymorphism influence IL-10 levels and may influence in the susceptibility to clinical malaria in Amazonian population.

## Abbreviations

SNP: Single nucleotide polymorphism
PCR: Polymerase chain reaction
DNA: Deoxyribonucleic acid
IFN-γ: Interferon-gamma
NO: Nitric oxide
TNF: Tumour necrosis factor
IL-10: Interleukin 10
iNOS: Enzyme nitric oxide synthase
NADPH: β-nicotinamide adenine dinucleotide phosphate
OR: Odds ratio
CI: Confidence interval
HWE: Hardy Weinberg equilibrium
EM: Expectation Maximization
E: Hope
M: Max
